# The Role of Alternative Polyadenylation in the Regulation of Subcellular RNA Localization

**DOI:** 10.3389/fgene.2021.818668

**Published:** 2022-01-14

**Authors:** Ankita Arora, Raeann Goering, Hei Yong G. Lo, Joelle Lo, Charlie Moffatt, J. Matthew Taliaferro

**Affiliations:** ^1^ Department of Biochemistry and Molecular Genetics, University of Colorado Anschutz Medical Campus, Aurora, CO, United States; ^2^ RNA Bioscience Initiative, University of Colorado Anschutz Medical Campus, Aurora, CO, United States

**Keywords:** alternative polyadenyaltion, RNA localization, RNA metabolism, 3′ UTR, RNA trafficking

## Abstract

Alternative polyadenylation (APA) is a widespread and conserved regulatory mechanism that generates diverse 3′ ends on mRNA. APA patterns are often tissue specific and play an important role in cellular processes such as cell proliferation, differentiation, and response to stress. Many APA sites are found in 3′ UTRs, generating mRNA isoforms with different 3′ UTR contents. These alternate 3′ UTR isoforms can change how the transcript is regulated, affecting its stability and translation. Since the subcellular localization of a transcript is often regulated by 3′ UTR sequences, this implies that APA can also change transcript location. However, this connection between APA and RNA localization has only recently been explored. In this review, we discuss the role of APA in mRNA localization across distinct subcellular compartments. We also discuss current challenges and future advancements that will aid our understanding of how APA affects RNA localization and molecular mechanisms that drive these processes.

## Introduction

Co-transcriptional maturation of pre-mRNA involves three steps - capping, splicing, and formation of a 3′ end by cleavage and addition of a poly(A) tail. For many transcripts, there are alternative cleavage and polyadenylation sites (PAS) that can generate multiple mRNA isoforms with different 3′ UTRs. This phenomenon, termed alternative polyadenylation (APA), generates distinct 3′ termini on mRNAs and thus allows for a single gene to create multiple transcripts, each with different 3′ UTR contents. These alternative 3′ UTR isoforms allow for inclusion or exclusion of *cis*-regulatory elements such as RNA binding protein sites and microRNA binding sites that then lead to changes in transcript abundance, stability, translation efficiency.

**FIGURE 1:**
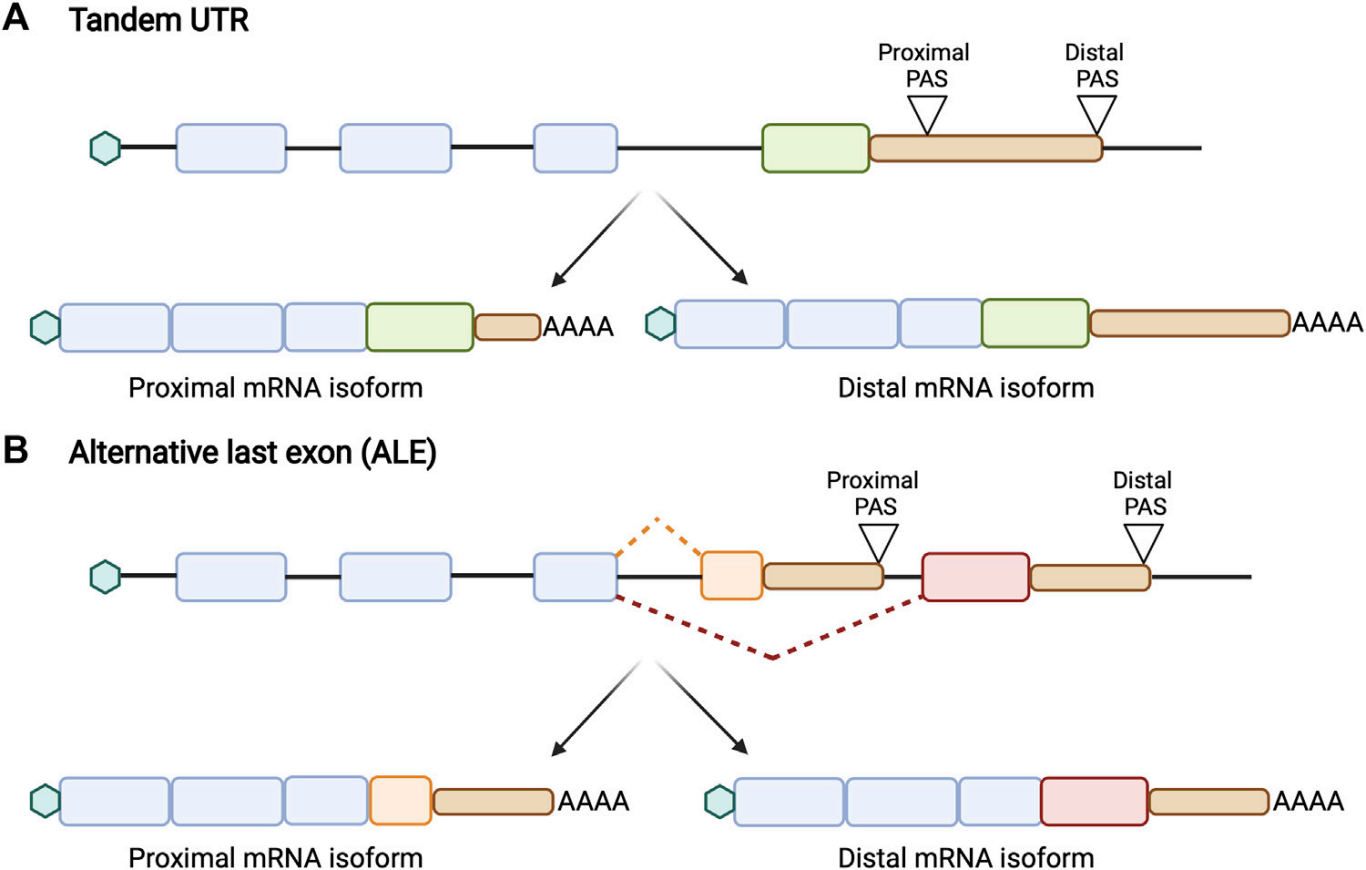
Schematic of two different forms of APA. **(A)** Tandem UTRs are characterized by the presence of an upstream proximal polyadenylation site (PAS) and a downstream distal PAS within the same terminal exon, generating multiple mRNA isoforms that differ in their 3′ UTR length without affecting the protein sequence. The use of the distal PAS leads to a longer 3′ UTR while the use of proximal PAS generates a shorter 3′ UTR. **(B)** Alternative last exons (ALEs) are characterized by the presence of multiple APA sites in different last exons. This results in producing both multiple mRNA and protein isoforms. The position of these ALEs is not correlated to its transcript length.

APA isoforms can generally be classified into two different forms. The first form, which we will define as “tandem UTRs”, occurs when multiple APA sites are present within the same terminal exon (Figure 1A). In this case, the use of upstream, gene-proximal cleavage sites always creates shorter transcripts than the use of downstream, gene-distal cleavage sites. The second form, which we will define as “alternative last exons” or “ALEs”, occurs when multiple APA sites are present in different terminal exons (Figure 1B). The position of these ALEs is not necessarily related to transcript length, and therefore it is incoherent to describe these events by their length. For ALEs, then, we will refer to the upstream PAS as the proximal site and the downstream PAS as the distal site.

The core 3′-end processing machinery is comprised of four main complexes: cleavage and polyadenylation specificity factor (CPSF), cleavage factor I (CFIm), cleavage factor II (CFIIm), and cleavage stimulation factor (CstF) (Shi et al., 2009). The polyadenylation signal (AAUAAA) is located 15–30 nucleotides upstream of the cleavage and PAS. The ​​WD repeat domain 33 and CPSF-30 subunits recognize the polyadenylation signal while CPSF-73 is the endonuclease that cleaves the pre-mRNA at the cleavage site between a cytosine/adenine (CA) dinucleotide. Once these complexes are assembled on the pre-mRNA, poly(A) polymerase (PAP) is recruited and stimulated by Poly(A) binding protein nuclear one to add poly(A) tail at the cleavage site [as reviewed in (Tian and Manley, 2017)].

The process of the complex formation is tightly regulated. Any small changes in the availability of these core factors affect 3′-end processing and can modify the expression of alternative 3′-UTR isoforms. In addition to the core 3′-end processing complex, several splicing factors such as U1 snRNP have been shown to regulate APA (Kaida et al., 2010; Berg et al., 2012) indicating that the splicing and polyadenylation mechanism are interconnected. A large number of other RBPs have shown to also play a role in APA regulation by blocking the binding of core 3′-end processing factors, or by facilitating the recruitment of core factors to the pre-mRNA [as reviewed in (Di Giammartino et al., 2011; Tian and Manley, 2017)]. A well-studied example is the ELAV (embryonic-lethal abnormal visual) protein, which causes 3′-UTR lengthening in *Drosophila* neurons by suppressing the use of proximal PAS (Hilgers et al., 2012; Oktaba et al., 2015).

Proteins are often asymmetrically distributed in spatially restricted subcellular compartments according to their respective functions. This protein localization in many cases is driven by prior localization of its cognate mRNA. Translation of these mRNAs within their specialized compartments facilitates rapid protein production allowing for timely temporal regulation. RNA localization therefore allows for an immediate, precise, and robust response to environmental conditions, and contributes to the function of diverse eukaryotic cell types including fibroblasts (Mili et al., 2008; Yamagishi et al., 2009), *Xenopus laevis* oocytes (Mowry and Melton, 1992; Deshler et al., 1997), *Drosophila melanogaster* embryos (Hachet and Ephrussi, 2004; Zimyanin et al., 2008), *Saccharomyces cerevisiae* (Long et al., 1997; Bertrand et al., 1998) and mammalian neurons (Cajigas et al., 2012; Goering et al., 2020). These asymmetrically distributed RNAs contribute to complex cellular functions. For instance up to 70% of mRNAs are expressed in distinct spatial patterns during *D. melanogaster* development (Lécuyer et al., 2007), and in many cases, as exemplified by the *osk* mRNA, the disruption of these localization patterns leads to developmental defects (Ephrussi and Lehmann, 1992; Lasko, 2012). In neurons, localization of mRNAs and their *in situ* translation in the dendritic spines contribute to synaptic plasticity and long-term memory (An et al., 2008; Lemieux et al., 2012). In axons, it is required for adapting in response to extracellular signals, especially during axon regeneration following an injury (Wang et al., 2007).

The localization of these RNAs is regulated by *cis*-acting sequence elements present mostly in the 3′ UTRs of the transcripts, often termed “zipcodes” (Jambhekar and Derisi, 2007; Meer et al., 2012). These zipcodes serve as a marker to target an RNA for transport to a specific subcellular location. These sequences are then identified by *trans*-acting RNA-binding proteins (RBPs) that bind with RNA to form ribonucleoprotein complexes (mRNPs) and mediate specific transport (Martin and Ephrussi, 2009; Buxbaum et al., 2015). However, some RBPs contribute to asymmetric mRNA localization through retention of transcripts in specific compartments. For example, an RBP Pumilio-2 (Pum2) is restricted to the cell body of developing neurons and contributes to maintaining cell polarity by confining mRNAs that contain the Pumilio binding element (UGUAHAUA, with H representing A, C, U, but not G) to the cell body (Martínez et al., 2019).

In recent years, it has been found that APA affects the majority of protein-coding genes. For example, over 70% of mammalian protein-coding genes encode multiple transcript isoforms derived from APA (Hoque et al., 2013). However, how APA affects subcellular RNA localization is only beginning to be understood. Since APA contributes to diversity of the transcriptome by generating multiple isoforms that differ in their 3′ UTRs and 3′ UTRs are hotspots for the regulation of RNA trafficking, APA has a huge potential to regulate RNA localization. Here, we review studies that highlight how APA affects RNA localization across various subcellular compartments and contributes to cellular functions.

### APA in Neurons

In highly polarized cell types like neurons, transcripts are often differentially localized between cell body and projection compartments (Cajigas et al., 2012). For some genes, APA isoforms are differentially localized between these compartments. This has been studied in-depth for two genes in two systems: rat *Impa1* and mouse *Bdnf*.

**FIGURE 2:**
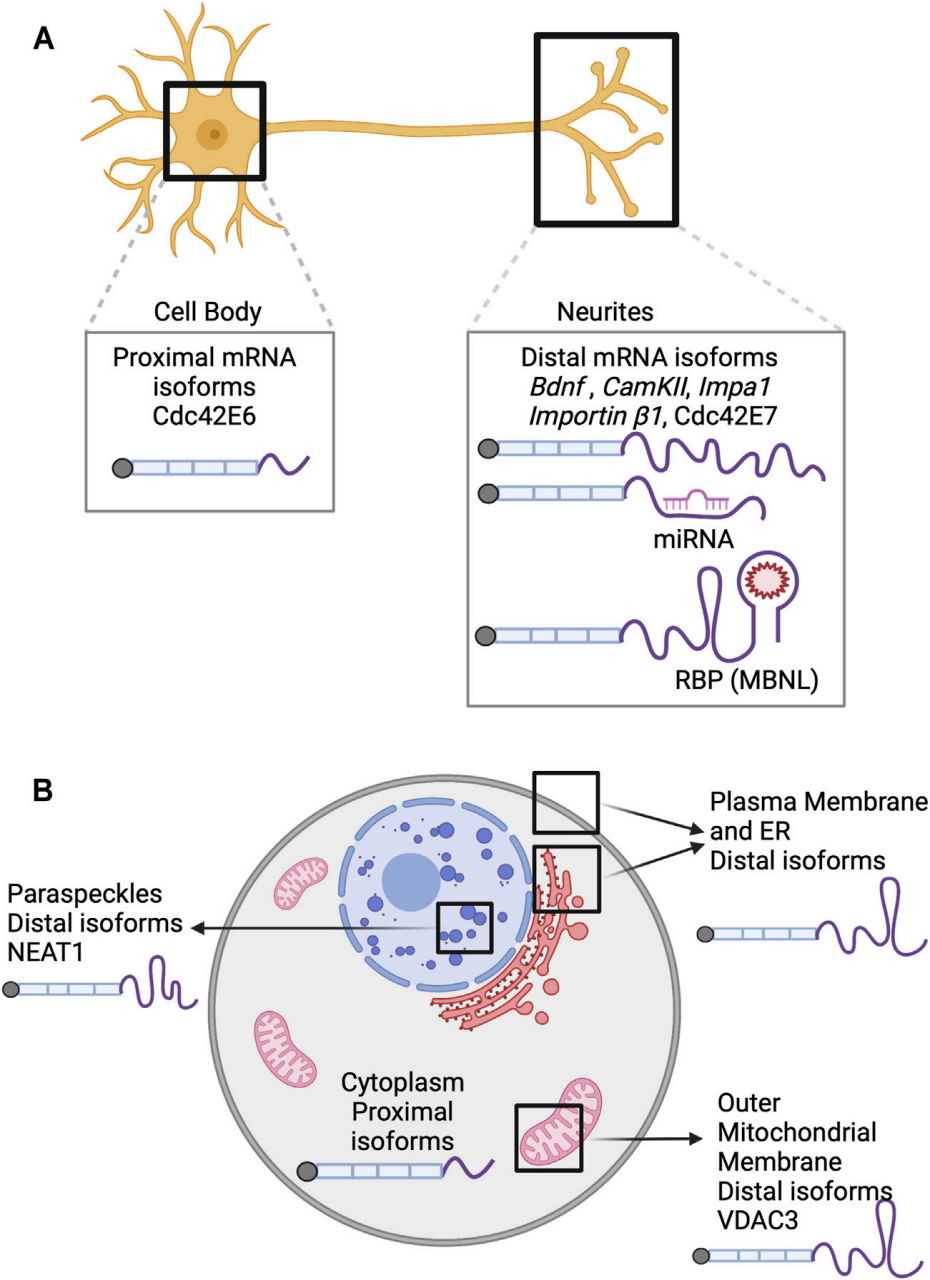
APA isoforms and their role in RNA localization. **(A)** In neurons, the mRNA isoforms that preferentially use the distal PAS have been shown to be localized in neurites while the isoforms that use the proximal PAS are enriched in the cell bodies. *Bdnf* (An et al., 2008), *CamKII* (Lemieux et al., 2012), *Impa1* (Andreassi et al., 2010) and *Importin β1* (Perry et al., 2012) are examples of tandem UTRs and thus, the distal PAS usage produces longer 3′ UTRs that allows increased binding sites for trans factors such as miRNA and RNA binding proteins. **(B)** Transcripts resulting from distal APA usage are enriched in the membrane-associated fraction. The VDAC3 (Michaud et al., 2014) isoform that uses the distal PAS harbors a *cis*-element that is sufficient to localize it to the outer mitochondrial membrane. The longer isoform of the lncRNA NEAT1(Modic et al., 2019) acts as a scaffold and localizes to paraspeckles. RBP: RNA binding protein, MBNL: Muscleblind-like proteins.

Rat IMPA1 protein is localized to axons. The *Impa1* 3′ UTR contains two polyadenylation sites. Usage of the more distal site creates a long 3′ UTR, leading to axonal localization of the transcript (Figure 2A). Transcripts that preferentially use the proximal PAS tend to remain in the soma (Andreassi et al., 2010). Thus, APA site choice directly influences transcript localization.

The mouse *Bdnf* gene also contains APA sites that create tandem 3′ UTRs. The section of 3′ UTR unique to the longer isoform is sufficient to localize GFP-encoding transcripts to neurites, where it is translated locally. Loss of distal PAS usage decreases *Bdnf* transcript localization to dendrites. This disruption also leads to greater dendritic spine density, but with smaller spine heads due to decreased spine trimming. Furthermore, the long 3′ UTR isoform is required for long term potentiation in dendrites, a necessity for synaptic plasticity. By modulating the localization of *Bdnf* mRNA, the APA of this transcript plays an important role in synaptic plasticity (An et al., 2008).

To expand the study of RNA localization in neuronal cells to a transcriptomic scale, multiple groups have used subcellular fractionation approaches in which cells are separated into cell body and projection fractions (Zivraj et al., 2010; Gumy et al., 2011; Taliaferro et al., 2016; Zappulo et al., 2017; Martínez et al., 2019; Arora et al., 2021a). RNA can be collected from these fractions and sequenced, giving information on the localization of thousands of transcripts at once. When this technique was applied to two lines of cultured mouse neuronal cells (N2As and CADs), it revealed that endogenous transcripts that use distal ALEs were significantly more likely to be localized to neuronal projections than to the cell body. Fusion of 3′ UTRs from proximal or distal ALEs to a reporter recapitulated this phenomenon, confirming that transcripts with 3′ UTRs derived from the distal ALE were sufficient for localization to neurites. The use of distal ALEs mostly led to inclusion of more binding motifs for RNA binding proteins in the 3′ UTR, including proteins in the Muscleblind-like (MBNL) family (Taliaferro et al., 2016). The association between distal PAS usage and localization of transcripts to projections of migrating cells was also observed in NIH3T3 and MDA-MB231 cells (Goering et al., 2021). Further, it was demonstrated that the use of distal PAS in both tandem UTR and ALE structures was associated with localization to cellular projections (Goering et al., 2021).

### APA Regulates Neuronal Signaling and Function

3′ UTR heterogeneity can influence which transcripts are enriched in different compartments of the mammalian hippocampus. In a study, 3′ end sequencing of RNA isolated through microdissecting of somata (cell body enriched) and neuropil (axon- and dendrite-enriched) layers from the rat hippocampus showed that localized transcripts contained strong preferences in APA site usage compared to non-localized transcripts. For example, dendrite-localized *CamKIIα* transcripts displayed increased distal PAS usage, favoring a longer 3′ UTR that harbors localization regulatory elements. Further, transcripts using distal PAS had increased stability, potentially enhancing their dendritic localization (Tushev et al., 2018).

Compartment-specific RNA localization has also been observed in mouse cortical neurons. For example, *Cdc42*, a small GTPase that controls the actin cytoskeleton and can influence cellular morphology. In the brain, *Cdc42* is responsible for regulating axon and dendrite outgrowth, dendrite arborization, and spinal development. APA of *Cdc42* RNA yields two isoforms with different last exons whose encoded proteins have distinct functions and localization in neurons. The proximal ALE isoform encodes CDC42E6, which localizes to the dendritic spines and is involved in their formation. On the other hand, the distal ALE isoform encodes CDC42E7, which has a role in axonogenesis (Kang et al., 2008; Yap et al., 2016). At the time, it was unknown if this differential protein localization was supported by differential RNA localization.

Later, using a combination of RNA-seq, 3′ end RNA-seq, Ribo-seq, and mass spectrometry, it was demonstrated that the alternative last exons of *Cdc42* act as important drivers for localization of both the RNA and protein isoforms to distinct compartments of the neuron. Namely, *Cdc42E6* RNA was found to be soma-enriched, and *Cdc42E7* RNA was neurite-enriched (Figure 2A). This differential enrichment in different compartments, accompanied by their local translation, contributes to maintaining neuronal polarity (Ciolli Mattioli et al., 2019).

APA is also an important regulator of axonal signaling, with localized translation of mRNAs playing a critical role in axonal maintenance and repair. *Importin β1* is thought to be a core component of the retrograde injury-signaling complex that utilizes local translation at injury sites in the axons and then relay stress back to the cell bodies. Two 3′ UTR variants of Importin RNA exist. These include a short isoform that uses the proximal PAS and is more prominent in cell bodies and a long isoform that uses the distal PAS and is more abundant in axons (Perry et al., 2012). Depletion of the distal APA isoform of *Importin β1* decreased axonal RNA localization. Further, in mice lacking the 3′ UTR of *Importin β1,* recovery from a peripheral nerve lesion was delayed. This suggests that an *Importin* APA-dependent transport mechanism may be responsible for timely axonal recovery.

Lastly, alternative 3′ UTRs have been shown to play an important role in neuronal responses to external stimuli and regulating the resulting gene expression changes. Using 3′ READS (Hoque et al., 2013), it was observed that a widespread shift towards proximal PAS usage and activation of intronic APA isoforms occurred 3 h post long term potentiation (LTP) in mouse hippocampal CA3-CA1 synapses (Fontes et al., 2017). For example, post-LTP Notch1 prefers use of proximal PAS leading to shortening of the 3′ UTR, and thereby loss of binding sites of miR-384-5p. This enables Notch1 to escape miRNA-mediated destabilization and translation repression, contributing to LTP maintenance and hippocampal synaptic plasticity. Since APA generates mRNA isoforms with different 3′ UTRs, which in turn are known to regulate RNA localization, these mRNA isoforms resulting from LTP induction may localize to distinct subcellular compartments and contribute to temporal regulation. This is in agreement with previous studies that explore the connection between use of proximal PAS and repression of miRNA-mediated silencing in other cell types such as during T-cell activation (Sandberg et al., 2008) and oncogenesis (Mayr and Bartel, 2009).

### APA and RNA Localization to Membranes

Similar to neuronal fractionation, where RNA localization is inferred from the RNA content of neurite and cell body fractions, RNA localization to membranes can be inferred from biochemical fractionations. Biochemical fractionations often yield three distinct fractions: cytosolic, insoluble (chromatin or cytoskeleton-associated), and membrane-associated, where components of the endoplasmic reticulum (ER), mitochondria and plasma membrane are found. RNA is isolated from each fraction, allowing for identification of localized transcripts (Jagannathan et al., 2011; Benoit Bouvrette et al., 2018). Further analysis can reveal if the 3′ end of a localized RNA is uniquely associated with efficient localization to the different fractions. RNA 3′ end choice impacts localization as longer isoforms can include additional RBP binding sites that modulate RNA localization where transcripts with more proximal APA lack the necessary RBP binding sites or localization elements.

General trends of RNA localization to membranes and 3′ end choice have been revealed by LABRAT, an APA quantification software (Goering et al., 2021). LABRAT analysis of biochemical fractionations derived from three different cell types (Benoit Bouvrette et al., 2018) revealed that longer (more distal APA) transcripts are enriched in the membrane-associated fraction (Figure 2B) (Goering et al., 2021). High agreement between 3′ end usage and membrane localization among three different cell types suggests a conserved mechanism of RNA localization to membranes that depends on APA status.

Because the ER comprises a large fraction of cellular membrane, it is assumed that most RNAs in the membrane-associated fraction originate from the ER. Surprisingly, RNAs localized to the membrane fraction by more distal APA usage are depleted for known peptide-encoded ER localization signal sequences (Goering et al., 2021). This suggests two separate mechanisms of RNA localization to the ER. One through signal sequences where RNAs are co-translationally localized by a nascent peptide signal and a second localization mechanism mediated by the transcript’s 3′ end. This argument is supported by the discovery of membrane proteins on the ER that can serve as receptors for binding mRNAs such as the positively charged p180 (Cui et al., 2012) and Astrocyte elevated gene-1 (AEG-1) (Hsu et al.,2018).

In order to increase the resolution and specificity to isolate ER-associated transcripts, polysome profiling was performed on the membrane associated fraction isolated from myoblasts. These translating RNAs are enriched in the ribosome coated rough ER where substantial protein synthesis occurs. ER-localized RNAs identified through this method were also found to be longer and have more distal APA usage (Figure 2B) (Cheng et al., 2021). Further, Cheng et al. analyzed these ER fractions in the presence and absence of translation inhibitors and categorized transcripts as having translation-dependent or translation-independent ER association.

Co-translationally ER localized RNAs are enriched for known ER localization signal sequences that create a nascent peptide signal recognized and localized by the signal recognition particle complex. While these translation-dependent ER localized RNAs tend to have longer 3′ ends and use more distal APA, this effect is accentuated when translation is inhibited. These results bolster the notion that two distinct mechanisms of RNA localization to the ER exist: co-translational localization, and translation-independent, 3′ end mediated localization. However, further experimentation with more precise techniques such as proximity labeling assays, like APEX-seq (Fazal et al., 2019) or HaLo-seq (Engel et al., 2021), would aid in further elucidating two separate mechanisms of RNA localization to the ER.

RBP binding to alternative 3′ UTRs is one of many mechanisms that can regulate RNA localization at the 3′ end isoform level (Figure 2A). When the RBPs of the MBNL family were depleted, distal APA isoforms became less associated with the membrane fraction (Wang et al., 2012). Experiments like these are needed to further understand the mechanisms that regulate the differential localization of APA isoforms to membranes.

In some cases, the localization of a protein depends on the APA isoform from which it was produced in an RNA localization-independent manner. The protein localization of CD47 was found to be dependent on the 3′ end choice of its RNA transcript (Berkovits and Mayr, 2015). It was further shown that while the short and long isoform of *CD47* have the same RNA localization and encode identical proteins, the resulting protein products are differentially localized to the ER and plasma membrane respectively. This localization mechanism, termed 3′ UTR-dependent protein localization, is mediated by a transfer of proteins bound to the 3′ UTR during translation to the nascent peptide determining both the protein’s distinct localization and cellular function.

In addition, only the longer isoform of *CD47* contains multiple AU-rich elements and binds to TIS11B to form membraneless organelles called TIS granules. These granules are intertwined with the ER creating a subcellular compartment called the TIGER domain that enables formation of specific protein-protein interactions that are dependent on 3′ UTRs of mRNAs (Ma and Mayr, 2018). These granules create mesh-like networks that require large-scale intermolecular RNA-RNA interactions to maintain their morphology (Ma et al., 2021). This highlights that alternative 3′ UTR isoforms can also influence the localization of mRNAs to membraneless compartments within the cell.

### APA-Mediated RNA Localization and its Role in Development

As cells become more specialized during development, APA plays a defining role in differentiation. Over the course of embryonic development in mice, mature, differentiated cells preferentially express transcripts that use distal APA sites while undifferentiated cells are more likely to express transcripts that use proximal APA sites (Ji et al., 2009). Using probes specific to the distal APA isoforms in Genechip arrays, it was found that during organogenesis, there is a global shift towards distal APA site usage. Specifically, using an experimental myogenesis model, it was demonstrated that the transcriptome of the differentiated myotubes was enriched in distal PAS usage compared to the transcriptome of undifferentiated myoblasts. Lastly, the authors used reporter constructs to show that actively dividing cells favored the expression of transcripts with the proximal PAS whereas differentiating cells preferred the expression of distal PAS. However, the biological significance of these longer transcripts remains unknown.

APA may play a role in differentiation by exposing or removing *cis*-acting elements that change RNA localization and thereby gene function. This was described for the case of inverted repeats of *Alu* (IR*Alus*). These structures are particularly abundant in the introns and 3′ UTRs of transcripts in the human genome (Chen et al., 2008). Some of these IR*Alus* are present in key genes that undergo APA, such as LIN28, a key regulatory factor in pluripotency. Shorter transcripts lacking these IR*Alus* tend to be found in the cytoplasm whereas transcripts with IR*Alus* are retained in the nucleus (Chen et al., 2008). Placing IR*Alu* elements in the 3′ UTR of a reporter transcript was sufficient to sequester that transcript in paraspeckles in the nucleus. The localization of IR*Alus*-containing transcripts differs based on cell state. IR*Alus* are sufficient to localize reporter constructs to paraspeckles in differentiated HeLa cells, but in naïve hESCs without nuclear speckles, IR*Alus-*containing transcripts are not retained in the nucleus (Chen and Carmichael, 2009). Taken together, these findings suggest not only that APA can influence RNA localization, but also that the localization elements can behave differently throughout development.

More recently, it has been found that APA changes the localization of *NEAT1* transcripts. The shorter, polyadenylated *NEAT1* gene does not form paraspeckles and is localized to the nucleosome whereas the full-length isoform is not polyadenylated, localizes around chromatin, and acts as a scaffold for paraspeckles (Figure 2B) (Modic et al., 2019). Localization of the *NEAT1* full-length transcript to speckles is critical for development of human or mouse ESCs through its interaction with TDP-43. TDP-43 regulates APA of many stem cell transcripts and prevents differentiation (Rot et al., 2017). Therefore, the APA of *NEAT1* indirectly allows for differentiation by sequestering TDP-43 to nuclear speckles and away from transcription sites.

Aside from regulating gene expression, APA of transcripts has also been shown to affect organelle maturation and growth. One example is the localization of voltage-dependent anion channels (VDAC) to the outer mitochondrial membrane (OMM) (Figure 2B). Loss of *VDAC3*’s distal APA site resulted in reduced localization of the transcript to the OMM by three-fold in *Arabidopsis thaliana* (Michaud et al., 2014). Additionally, the distal APA isoform of *VDAC3* retains a *cis*-element that is sufficient to localize an un-related mRNA to the OMM. Localization of *VDAC3* RNA to mitochondria directly correlates with mitochondria size and number. While only a single example, these findings suggest APA can directly regulate organelle development.

## Conclusion and Perspectives

RNA localization as a post-transcriptional regulatory process is highly prevalent across species. mRNA localization is mediated by *cis*-elements called zipcodes, most often present in its 3′ UTR. Given this crucial role of 3′ UTR in mRNA transport, the formation of alternate 3′ UTR isoforms can serve as an important mechanism to create diverse subcellular RNA localization patterns and ultimately, contribute to cellular physiology. However, we still lack a detailed understanding of how the majority of the RNAs are transported to their destination. For instance, while thousands of localized transcript have been identified in projections of neuronal cells, we only know handful of examples where the corresponding *cis*-elements and *trans*-factors that govern its transport are known. This lack of known zipcodes hinders our ability to unambiguously define many cases where one 3′ UTR isoform contains a zipcode while another doesn’t.

While we have an extensive knowledge of core processing factors that regulate APA, the transcript-specific RBPs that regulate differential localization of APA isoforms remain to be identified. The advent of transcriptome-wide RBP/RNA interaction identification methods such as RIP-seq, CLIP-seq, HITS-CLIP, and eCLIP has enabled high-resolution mapping of RBP-RNA binding interactions (Lee and Ule, 2018). However, the use of these techniques to study APA and its effects on localization is still limited, again largely due to our lack of knowledge of sequences that regulate RNA localization.

Traditionally, RNA localization has been studied using imaging techniques such as, Fluorescent *in-situ* hybridization (FISH) (Bertrand et al., 1998), single-molecule FISH (Raj et al., 2008) and genetically encoded RNA reporters (MS2, Pumilio, PP7) (Urbanek et al., 2014). However, these imaging approaches focus on studying one transcript at a time and are not easily amenable to high-throughput analysis, hindering the ability to identify transcriptome-wide trends.

One approach toward bridging these knowledge gaps is the use of large scale screens. For example, one such RNAi screen surveyed 489 RBPs to identify regulators of PAS usage (Jenal et al., 2012). Similarly, massively parallel reporter assays have recently been used to identify sequence elements that regulate RNA localization (Arora et al., 2021b; Mikl et al., 2021; von Kügelgen et al., 2021). The synthesis of these large scale datasets has the potential to identify more cases in which the regulation of APA and RNA localization are explicitly linked.

In the future, implementing long-read sequencing methods to directly sequence poly(A) RNAs without fragmentation offer higher accuracy and sensitivity to detect and quantify APA isoforms (Sharon et al., 2013; Feng et al., 2015; Shah et al., 2021). Additionally, using machine learning models to predict novel PAS (Li et al., 2021; Lusk et al., 2021) and improvements in quantification methods to analyze different isoforms in spatially restricted transcriptomes will greatly improve our understanding of the APA regulation and how it affects RNA localization. In recent years, proximity-based labeling methods (Fazal et al., 2019; Engel et al., 2021) have enabled the study of RNA localization in diverse cell types. These advancements combined may further increase the number of examples where APA affects RNA localization and thereby allow understanding of generalized mechanisms by which the two are coregulated.
